# A Phenomenological Theory of Spatially Structured Local Synaptic Connectivity

**DOI:** 10.1371/journal.pcbi.0010011

**Published:** 2005-06-24

**Authors:** Bagrat Amirikian

**Affiliations:** Brain Sciences Center, Veterans Affairs Medical Center, Minneapolis, Minnesota, United States of America, and Department of Neuroscience, University of Minnesota, Minneapolis, Minnesota, United States of America; University College London, United Kingdom

## Abstract

The structure of local synaptic circuits is the key to understanding cortical function and how neuronal functional modules such as cortical columns are formed. The central problem in deciphering cortical microcircuits is the quantification of synaptic connectivity between neuron pairs. I present a theoretical model that accounts for the axon and dendrite morphologies of pre- and postsynaptic cells and provides the average number of synaptic contacts formed between them as a function of their relative locations in three-dimensional space. An important aspect of the current approach is the representation of a complex structure of an axonal/dendritic arbor as a superposition of basic structures—synaptic clouds. Each cloud has three structural parameters that can be directly estimated from two-dimensional drawings of the underlying arbor. Using empirical data available in literature, I applied this theory to three morphologically different types of cell pairs. I found that, within a wide range of cell separations, the theory is in very good agreement with empirical data on (i) axonal–dendritic contacts of pyramidal cells and (ii) somatic synapses formed by the axons of inhibitory interneurons. Since for many types of neurons plane arborization drawings are available from literature, this theory can provide a practical means for quantitatively deriving local synaptic circuits based on the actual observed densities of specific types of neurons and their morphologies. It can also have significant implications for computational models of cortical networks by making it possible to wire up simulated neural networks in a realistic fashion.

## Synopsis

Each neuron communicates signals via synaptic connections simultaneously to several hundreds of neighboring neurons forming a synaptic circuit. Determining the pattern of synaptic connections between local neurons is crucial for understanding a specific cortical function implemented by a synaptic circuit. The connectivity between a pair of neurons is affected by their axonal/dendritic morphologies and relative spatial locations. Although neuroscientists have precise tools to measure neuronal activity caused by the flow of signals between circuit neurons, there are still considerable difficulties in the direct experimental measurement of local synaptic connectivity, which actually determines the underlying activity. This paper presents a theoretical approach to synaptic connectivity accounting for the morphologies of pre- and postsynaptic neurons and providing the average number of synaptic contacts formed between them as a function of their relative locations. An important aspect is the decomposition of the complex structure of an axonal/dendritic arbor into a small number of basic structures. The theory is in very good agreement, within a wide range of cell separations, with empirical data on axonal–dendritic contacts of pyramidal cells and somatic synapses formed by the axons of inhibitory interneurons. The current approach can provide a practical means for quantitatively deriving local synaptic circuits based on the actual observed densities of specific types of neurons and their morphologies.

## Introduction

Unraveling intrinsic cortical circuitry—the pattern of synaptic connections between neurons within a local region—still is one of the most difficult challenges faced by researchers in neuroscience. The structure of intrinsic circuitry is the key to understanding cortical function and how neuronal functional modules such as cortical columns are formed [1–9]. The central obstacle to progress is the quantification of synaptic connectivity among different types of cells. The connectivity between a pair of pre- and postsynaptic neurons is affected by their axonal and dendritic morphologies, and their relative spatial locations. While neocortical neurons have diverse morphology, the number of basic morphologically distinct types of neurons is only of an order of 10^1^ [10], and it is much smaller than the number of neurons themselves, which is of an order of 10^10^ for the human cortex [2]. This fact engenders an intuition that the problem of deciphering local cortical circuitry in such a huge network could be theoretically tractable. Indeed, the number of possible morphologically different types of pre- and postsynaptic cell pairs that it might be necessary to consider is relatively small, of an order of 10^1^ × 10^1^ = 10^2^. Furthermore, instead of treating each cell pair individually, synaptic connectivity can be averaged over a whole ensemble of pairs in which all pre- and postsynaptic neurons have their respective underlying morphologies. An orderly relationship found by Sholl [11] describing apparently random branching patterns of dendrites of individual pyramidal and stellate cells inaugurated the idea of a statistical approach to the problem of synaptic connectivity, and motivated subsequent experimental and theoretical studies that have been carried out in this spirit.

To form a synaptic contact it is necessary that the presynaptic axon comes to a close spatial apposition to a certain cellular site (dendrite, soma, axon hillock, etc.) located on the postsynaptic neuron, establishing a physical contact. For the purpose of synaptic connectivity, it is useful to distinguish morphological features of neurons at large and small spatial scales. Large-scale features, such as the characteristic shape and size of a volume occupied by axonal/dendritic ramifications, set the limits of spatial separation between a pair of neurons within which they can potentially establish physical contacts and thus affect how the synaptic connectivity changes as a function of their relative positions.

Small-scale morphological features, such as the length and local curvature of axonal branches, can reveal cellular site specificity of synaptic contacts. The question of whether synaptic connectivity is random or specific has been addressed in several studies [3,6,12–18]. It has been noticed that small-scale features of the axons of most neurons (70%–80%), which are excitatory pyramidal cells [2], have a remarkable property: axon branches usually extend as straight lines for fairly long distances. This, as well as other observations, supports the idea that pyramidal cell axons make contacts in a nonspecific fashion, at random sites on the postsynaptic cell dendrites, when they are encountered by chance along the axon path [15,19].

Morphological properties of the remaining (20%–30%) neocortical cells, which are mostly inhibitory interneurons [2], are more intricate. It has been shown [18] that their axons have significantly smaller branch lengths, and the trajectories are considerably curved. Such geometrical characteristics suggest [18] that inhibitory interneuron axons are attracted to specific targets scattered around, in contrast to pyramidal cell axons, which shoot straight through neuropil for long distances. Indeed, interneurons can be divided into several types on the basis of what specific cellular sites on the postsynaptic neuron their axons are targeting; for example, there are dendrite- and tuft-targeting, dendrite-targeting, proximal dendrite- and soma-targeting, and axon-targeting interneurons [10].

Quantitative studies of synaptic connectivity are usually restricted to the case of nonspecific connections, when geometries of axons and dendrites are assumed to be mutually independent [15,19–23]. They can be divided basically into two categories. Studies in the first category make a number of specific assumptions about the morphologies of pre- and postsynaptic cells and then estimate synaptic connectivity between them [19–21,24]. Although these assumptions are relatively loosely related to the actual morphological structures of cells and to a certain extent are dictated by the convenience of analytical considerations, studies using this kind of approach offer a general framework for the treatment of the problem of synaptic connectivity and provide a broad view of the underlying picture. Studies in the second category, instead of making series of assumptions, use actual three-dimensional (3D) ramification patterns of reconstructed neurons and then estimate synaptic connectivity, supposing that the observed morphologies of presynaptic axons and postsynaptic dendrites are independent [22,23]. Although these kinds of studies are labor intensive, and provide a narrower view, being limited to a specific type of pre- and postsynaptic pair, they are quantitatively much more precise since the morphologies of real neurons are used for the prediction of connectivity.

In this paper I present a theoretical model of synaptic connectivity that strives to bring the strong aspects of each of these two different approaches into a single framework. For example, it takes into consideration large-scale morphological features of pre- and postsynaptic cells. However, it does not require 3D reconstructions of neurons; the necessary structural parameters of the underlying arbors can be directly estimated from plane, two-dimensional (2D) drawings of axons and dendrites. Importantly, such drawings are already available in literature for many different types of neurons. On the other hand, this theory does not explicitly consider small-scale morphological features of arbors but rather, in the spirit of several previous studies [15,19,25], introduces the notion of a synaptic density field. Making reasonable assumptions about the general structure of synaptic fields of axons and dendrites, I derived an expression for the average number of synaptic contacts formed between a pair of cells of given morphological types as a function of their relative locations in 3D space. To evaluate the impact of these assumptions, the theoretical number of contacts was compared with the number of contacts estimated empirically. I found that, within a wide range of cell separations, the theory is in very good agreement with empirical data on (i) axonal–dendritic contacts of pyramidal cells [22,23] and (ii) somatic synapses formed by the axons of inhibitory interneurons [26].

## Results

### Theoretical Framework

Synaptic connectivity between cortical neurons takes place at short-range (<10^3^ μm) and long-range (>10^3^ μm) scales [2,3]. The former is due to the local—with respect to the cell soma—ramifications of axonal and dendritic processes, whereas the latter is mediated by horizontally running axons and dendrites in cortical layers, and by axons of pyramidal cells leaving the cortex for the white matter and reentering it at distant locations. The present consideration deals only with local intracortical interactions. My approach is inspired by several previous quantitative studies of cortical connectivity [3,15,20,21,23,25].

#### Synaptic density field.

Suppose that one is able to record the spatial position of each synapse formed by the axon branches of a given presynaptic cell belonging to a particular morphological type *μ*_A_. Let us mark all recorded synaptic sites by dots and place this cell into a 3D coordinate system, so that its soma is at the origin of the system. The presynaptic sites will then form a dispersed cloud of dots distributed in a certain way relative to the origin. Let us now pick up another nearby cell of the same type *μ*_A_, record the spatial positions of all synapses formed by the axon branches of that cell, and then place it into the same coordinate system, again aligning its soma with the origin. Since the distribution of synaptic sites of the second cell is unlikely to be exactly the same as that of the first one, the number of dots would nearly double. However, given that both cells are of the same morphological type and are from the same cortical neighborhood, one would expect that the two dot patterns would be similar. These hypothetical experiments can be repeated many times. As the number of contributing cells increases, the dot clouds will become more and more dense, leading to a formation of a certain structure.

One can describe this structure in terms of the volume density of dots—synaptic sites—averaged over a large number of contributing cells of the same morphological type *μ*_A_. Such ensemble averaging defines the synaptic density field 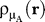 as the expected density of synapses formed by the axon ramifications of a single cell at spatial location **r** relative to the cell soma. Specifically, consider an element of volume Δ*V* with coordinate **r**. Let 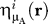 be the number of synaptic sites on the axon branches of cell *i* (*i* = 1, 2,…*n*_A_) within the volume Δ*V*. The contribution of neuron *i* to the synaptic density field at location **r** is then given by 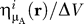 . Correspondingly, the sum of individual contributions from the entire ensemble yields the underlying density field 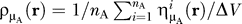 . In the framework of this approach, observed spatial distributions of synapses of different cells, even if belonging to the same morphological type, are random realizations of a certain structure determined by the underlying morphology of axons *μ*_A_. Conversely, synaptic density field 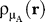 is the average of all possible realizations of the distributions of synaptic sites of individual cells:


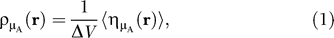


where 〈·〉 indicates ensemble averaging.

The consideration above can be carried out, likewise, for synapses formed on the dendrite branches of postsynaptic cells. This would result in a corresponding synaptic density field 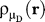 determined by the dendrite morphology *μ*_D_.

#### Synaptic contacts between a pair of cells.

Consider now a pair of cells separated by a displacement vector **d** pointing from the soma of the presynaptic cell with the axon morphology *μ*_A_ to the soma of the postsynaptic cell with the dendrite morphology *μ*_D_. Assume that the locations of all synaptic sites (including synapses formed with other neurons) on the presynaptic cell axon and the postsynaptic cell dendrite are recorded. Let us align the origin of the coordinate system with the soma of the presynaptic cell. Then the postsynaptic cell coordinate is **d**. One may pick up now another nearby pre- and postsynaptic cell pair composed of the same types of neurons separated by the same displacement **d**, record spatial positions of all synaptic sites on the presynaptic axon and postsynaptic dendrite, and place the pair into the same coordinate system. In the spirit of the single cell consideration above, this procedure can be repeated many times, giving rise to a large ensemble of contributing cell pairs. In such pairs, all presynaptic cells belong to the morphological type *μ*_A_. Likewise, all postsynaptic cells belong to their own morphological type, *μ*_D_. For a given cell pair *i* (*i* = 1, 2,…*n*), the number of synaptic sites on the axon branches of the presynaptic cell in the element of volume Δ*V* at location **r** is given by 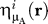 , whereas the number of synaptic sites on the dendrite branches of the postsynaptic cell at the same location is given by 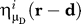 . It is assumed that Δ*V* is small enough, so that within this volume each individual cell may have at most one synaptic site, i.e., 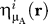 and 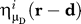 are either zero or one. If cells in the pair form a synaptic contact in Δ*V,* then the synaptic sites of both cells are present in this volume, i.e., 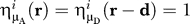 , and share a common spatial location. The opposite, however, is not always true. Specifically, if both cells in the pair do have synaptic sites in Δ*V,* it does not necessarily mean that there is a synapse between them. Indeed, these sites could form synapses with other cells that have axonal or dendritic ramifications in Δ*V*. Let 
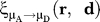
 be the proportion of pairs that form a synaptic contact in Δ*V* relative to the total number of pairs in which both cells have synaptic sites in the volume Δ*V*. The ensemble average of the volume density of synaptic contacts at location **r** between cell pairs separated by displacement **d** is then given by


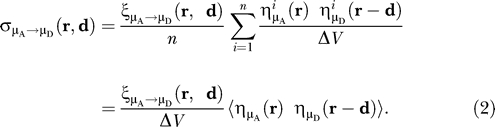


In the framework of this approach, 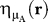 and 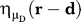 can be considered as random variables, whereas 
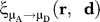
 can be treated as the probability of synaptic contact at location **r** between pre- and postsynaptic cells given that each of them has a synaptic site in the element of volume Δ*V*.

I now make two simplifying assumptions. First, I assume that 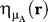 and 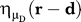 are distributed independently and, therefore, the average of their product is equal to the product of their averages:


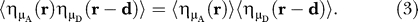


This means that, at any given spatial location **r**, the occurrences of synaptic sites on the axonal and dendritic ramifications of, correspondingly, the pre- and postsynaptic cells are independent from each other. This is true when the interaction (repulsion or attraction) forces between the axons and dendrites are relatively small and could be neglected. Second, I assume that 
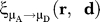
 , the probability of forming a synaptic contact, is determined by the likelihood of spatial proximity of the pre- and postsynaptic branches in Δ*V,* and that the rate of synapse formation (given that such a close spatial apposition has been established) is constant along the axon and dendrite branches and is independent of the type of morphologies *μ*_A_ and *μ*_D_. This can be formally expressed as


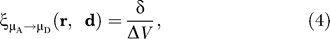


where *δ* is a free parameter representing the characteristic volume to be shared by the axon and dendrite segments in order to form a synapse.

Using equations 3, 4, and 1, the average density of synaptic contacts 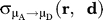 given by equation 2 can be then written as


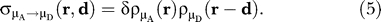


Integrating equation 5 over the entire space one can obtain the average number of synaptic contacts between a pair of cells as a function of the displacement **d**:


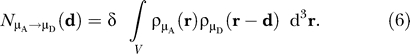


I assume that at any given spatial location, the ensemble distribution of the number of synaptic contacts within an element of volume Δ*V,* in which 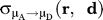 could be considered constant, follows a Poisson distribution with the mean 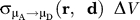 . Assuming that the distributions at different spatial locations are independent from each other (see, however, [23]), the total number of synaptic contacts, which is the sum of the local contributions, will also be distributed according to Poisson, with the mean 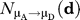 **,** as given by equation 6. Although observed distributions of the number of synapses per connection could be non-Poisson [27,28], for the purpose of the present consideration a Poisson distribution is a reasonably good approximation, and has been used in previous studies of synaptic connectivity [21,23].

### Structure of Synaptic Density Fields

The evaluation of the average number of synaptic contacts 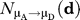 requires knowledge of the synaptic density fields 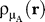 and 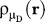 . To make the problem theoretically tractable, I introduce a number of simplifying assumptions about the general structure of 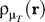 , where *T* designates either an axonal (*T* = A) or dendritic (*T* = D) tree.

#### Assumption 1.

Synaptic density fields have cylindrical symmetry. The axis of symmetry traverses the cell soma and is oriented vertically, orthogonal to the cortical layers. Thus, the density is isotropic in the horizontal dimension, parallel to the layers. This assumption is motivated by the laminated structure of the cortex [2]. Indeed, although along the vertical dimension, across the layers, cortical physical properties are heterogeneous and may change dramatically (density of cells, type of cells, etc.), in the horizontal dimension, within a local region, the properties are much more homogeneous and isotropic. Correspondingly, while axonal and dendritic arborizations of experimentally reconstructed cells may exhibit certain anisotropy in the horizontal dimension [26], I assume that apparent asymmetries shown by individual cells are averaged out when a large ensemble of cells of the same morphological type is considered, so that the ensemble averaged distribution of synapses is cylindrically symmetrical.

#### Assumption 2.

The arborization structure of a given morphological type *μ_T_* can be broken down into a number of basic structures—elementary clouds. The center 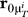 of an individual cloud 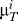 is located on the axis of symmetry at a certain distance from the cell soma. Accordingly, the synaptic density field representing the arborization *μ_T_* is a superposition of the density fields 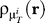 of the constituting clouds:


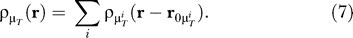


**Figure 1:**
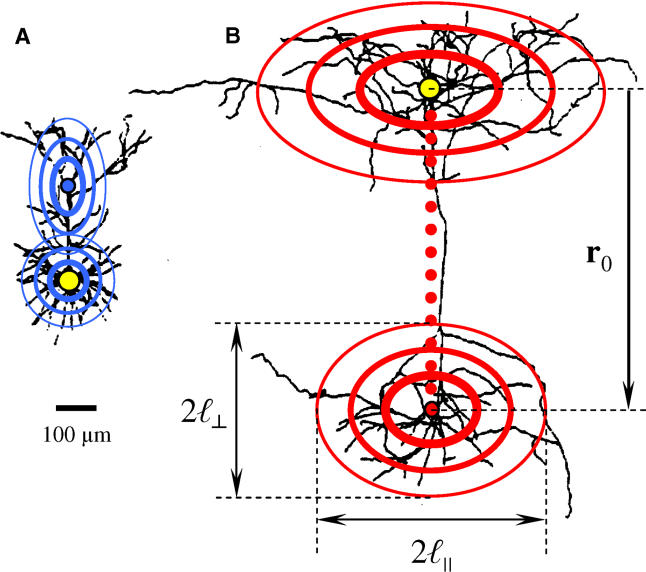
Decomposition of the Complex Structure of Arbors into Elementary Synaptic Clouds Synaptic density field of each cloud is illustrated by a set of concentric ellipsoids of different weights. An ellipsoid represents the equal-synaptic-density surface, whereas its weight represents the magnitude of the density. The outer ellipsoid, in addition, encloses the spatial extent of cloud ramifications. Yellow dots depict cell somata. The horizontal ℓ_||_ and vertical ℓ_⊥_ dimensions of one of the clouds as well as the displacement **r**_0_ of its center from the soma are shown. (A) A drawing of the dendritic arbor typical for L3 pyramidal neurons. (B) A drawing of the axonal arbor typical for L2 pyramidal neurons. The drawings of arbors are based on data representations in [22] by kind permission of B. Hellwig.

Likewise, the average total number of synaptic contacts between a pair of cells is the sum of the contributions 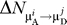 from individual axonal–dendritic cloud pairs:


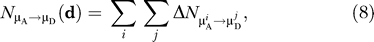


where


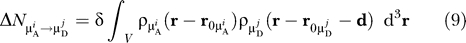


(cf. equation 6). This assumption is based on observations that axonal and dendritic morphologies of cortical cells often show distinct branching patterns that are well segregated in space. Figure 1 exemplifies this point. For example, a drawing of the axonal arbor [22] that is typical for layer 2 (L2) pyramidal neurons illustrates that in addition to a dense arborization of collaterals around the cell body, the axons also ramify extensively in deeper layers of the cortex (Figure 1B). It is natural, therefore, to describe the axonal arborization structure of these neurons as a superposition of two distinct clouds.

#### Assumption 3.

Equal synaptic-density surfaces of a given elementary cloud form a continuum of concentric, similar ellipsoids that are aligned at the cloud center. This assumption is motivated by the observation that the contours of the spatial spread of axonal and dendritic clouds often have an ellipsoidal shape (Figure 1). Because of the assumption of cylindrical symmetry, the ellipsoids revolve around the axis of symmetry. The shape of ellipsoids, oblate or prolate, depends on the actual spatial pattern of the ramifications. For example, the shape of the axonal clouds shown in Figure 1B can be approximated by oblate ellipsoids. On the other hand, the oblique apical dendrites of pyramidal cells can be well approximated by prolate ellipsoids, and the basal dendrites by spheres (Figure 1A).

#### Assumption 4.

Synaptic density falls off exponentially along the longitudinal and transverse axes of the ellipsoids. Specifically, I assume that the elementary cloud density field is given by


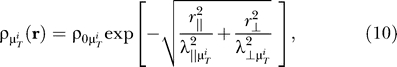


where 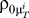 is the synaptic density at the center of the cloud 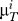 ; 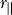 and 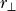 , correspondingly, are the longitudinal (parallel to the cortical layers) and transverse (perpendicular to the layers) components of the vector 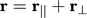 originating from the cloud center; and finally, 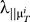 and 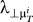 are the longitudinal and transverse space constants characterizing the rate of the density decay in the horizontal and vertical dimensions, respectively.

Such a choice of the density field function is motivated by the work of Sholl [11], who has measured the number of dendritic processes crossing the unit area on a sphere centered at the cell soma. Sholl has shown that for the dendritic systems of the stellate and pyramidal neurons in the striate and motor areas of the cat this number decays exponentially as a function of the sphere radius. Assuming that synapses are distributed uniformly randomly along the cell processes [15,29,30] the exponential law would also be applicable to the density of synapses received by an individual cell. I further postulate that a similar relationship holds for axonal processes.

Given that synaptic density fields are determined by equation 10, one can evaluate from equation 9 the average number of synaptic contacts between a specific pair of axonal 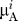 and dendritic 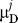 clouds. To simplify the calculations, it is convenient to introduce dimensionless variables:


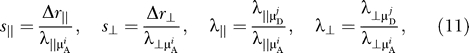


where 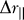 and 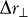 are, correspondingly, the longitudinal and transverse components of the displacement vector 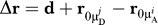 connecting the cloud centers. In general, the triple integral (equation 9) cannot be evaluated analytically. It can be reduced, however, to a one-dimensional integral (see Materials and Methods) that is easy to evaluate numerically:


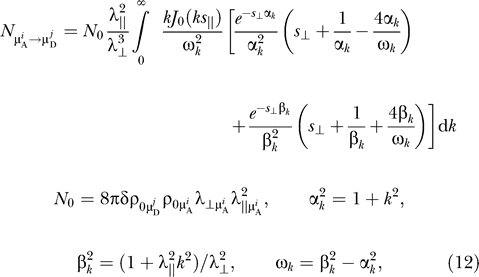


where *J*_0_ (*x*) is the Bessel function of order zero. In a special case, when 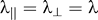 , i.e., the interacting axonal and dendritic clouds have similar shapes (not necessarily spherical), the integral (equation 9) can be evaluated analytically (see Materials and Methods):


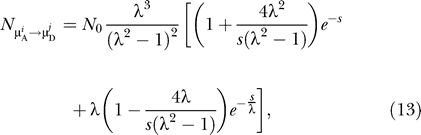


where 
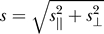
 .

### Comparison with Experiments

The central point of the present theory is to reduce the detailed picture of the complex branching patterns of axonal/dendritic processes and the locations of individual synapses along them to a simple but adequate (for the explanation of local synaptic connectivity) representation that is described by a small number of phenomenological parameters. To that end, the synaptic field of a given arbor is represented as a superposition of the synaptic fields of elementary clouds. For each elementary cloud 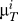 there are three structural parameters ( 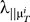 and 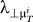 , characterizing the spatial spread of the cloud in the horizontal and vertical dimensions, respectively, and 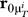 , defining the displacement of the cloud center from the cell soma) and one magnitude-scaling parameter ( 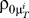 , describing the synaptic density at the cloud center). The impact of the underlying simplifying assumptions on the capacity of the theoretical model to quantify cortical synaptic connectivity can be assessed by comparing the predictions of the theory with experimental data. Below, using quantitative studies of synaptic connectivity carried out elsewhere, I consider three examples revealing the adequacy of the theory and illustrating how the above parameters can be evaluated from experimental data. Although in these studies only physical, not synaptic, contacts were directly [26] or indirectly [22,23] estimated from experimental data, they all presumed that physical connectivity is a good approximation of synaptic connectivity if the formation of a synaptic contact from an already established physical contact is a random and nonspecific process with a certain fixed rate.

#### Example 1: Pyramidal neurons in L2 and L3 of rat visual cortex.

Here I exploit Hellwig's work [22] in which axonal and dendritic arborizations of four L2 and four L3 pyramidal neurons of rat visual cortex were 3D reconstructed with the aim of estimating local connectivity from morphologies *μ*_A_ and *μ*_D_ of pre- and postsynaptic cells. This aim was achieved by counting the number of physical contacts between axonal and dendritic branches in a pair of reconstructed cells positioned at a certain distance from each other. The distance was varied by shifting the postsynaptic cell along an axis parallel to the coronal plane and the cortical surface, beginning from the maximum overlapping position in which a separation 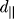 between the cell somata along the shifting axis was zero to a position in which 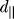 was 500 μm.

**Figure 2:**
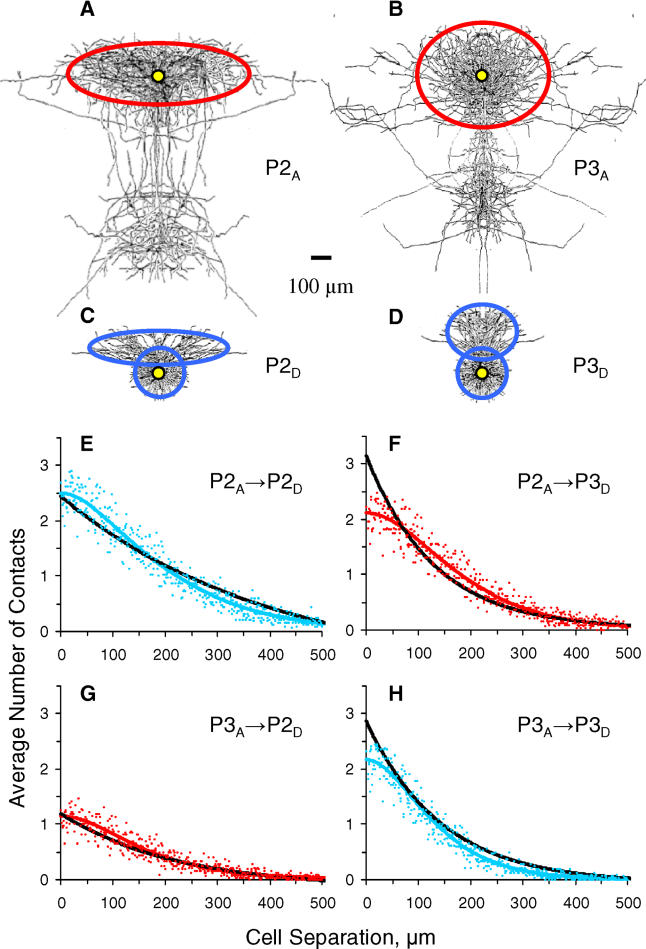
Connectivity between Pyramidal Neurons in L2/L3 of Rat Visual Cortex (A–D) Images representing the average structures of axons (A and B) and dendrites (C and D) of pyramidal neurons originating from L2 (A and C) and L3 (B and D). Yellow dots depict cell somata. Ellipsoids capture the spatial extent of the synaptic clouds identified from these images. The dimensions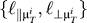 and the displacement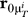 μ of each cloud were measured as illustrated in Figure 1B. The images were created using dendritic and axonal arborization drawings based on data representations in [22] by kind permission of B. Hellwig. (E–H) Average number of contacts between pre- and postsynaptic neurons as a function of the distance between them. The type of axonal–dendritic connection is shown on each plot. Empirical curves [22] are plotted in black. Fitted theoretical curves are plotted in blue and predicted curves are plotted in red. Dots show stochastic variations in the theoretical number of contacts.

Based on the layer of origin of the cell soma, Hellwig distinguished two different types of axons and two different types of dendrites. I designate them as P2_A_ and P3_A_ for the axons and P2_D_ and P3_D_ for the dendrites of neurons in L2 and L3, respectively (Figure 2A–2D). Thus, given that *μ*_A_ = {P2_A_, P3_A_} and *μ*_D_ = {P2_D_, P3_D_}, there can be four different types of pre- and postsynaptic cell pairs, resulting in four different types of axonal–dendritic connections *μ*_A_ → *μ*_D_.

For each type of axon and dendrite there were four 3D reconstructions. By averaging over 32 possible combinations of pre- and postsynaptic cell pairs of the same *μ*_A_ → *μ*_D_ type separated by a given distance (4 axons × 4 dendrites × 2 positions along the shifting axis resulting in the same separation), Hellwig [22] obtained empirical relationships 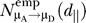 describing the average number of contacts between pyramidal neurons in L2 and L3 rat visual cortex as a function of horizontal cell separation 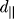 (Figure 2E–2H, black curves). To understand how well the present theoretical model can capture these empirical relationships, I adopted the following procedure.

First, I visualized average spatial structures of the reconstructed axons and dendrites. To that end, individual arborization drawings based on data representations in [22] were mirror reflected about the vertical axis. Then, the original and reflected drawings for all four arbors of a given type *μ_T_* (*μ_T_ =* {P2_A_, P3_A_, P2_D_, P3_D_}) were laid on top of each other so that the positions of all somata were aligned. The resulting image represented the average spatial structure of the underlying arbor type *μ_T_* (Figure 2A–2D). Note that this procedure made the average arbor images obtained from a relatively small experimental sample symmetric and implemented the assumption of the theory that synaptic density fields have cylindrical symmetry.

Second, visually inspecting these images, I identified elementary clouds of axons and dendrites, and enclosed them in distinct ellipses capturing the spatial extent of individual cloud ramifications (Figure 2A–2D). The morphologies of both P2_D_ and P3_D_ dendrites were described by two clouds corresponding to basal and apical ramifications represented by a sphere and an oblate ellipsoid, respectively (Figure 2C and 2D). The structures of P2_A_ and P3_A_ axons were described by single clouds corresponding to a dense arborization of collaterals around the cell body and were represented by oblate ellipsoids (Figure 2A and 2B). The clouds formed by the extensive branching of P2_A_ and P3_A_ axons at deeper layers were disregarded in the present consideration. The point is that they are well separated from the P2_D_ and P3_D_ dendrites that ramify in the upper layers and, therefore, their contribution to the connectivity between L2/L3 pyramidal neurons is negligible. Altogether, six distinct clouds were used to describe the two types of neuron morphologies. For each individual cloud 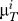 (*i* = 1 for *μ*_A_ and *i* = 1,2 for *μ*_D_) the horizontal, 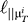 , and vertical, 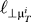 , semi-axes of the corresponding ellipse as well as the position of its center relative to the cell soma, 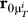 , were estimated from the drawings in Figure 2A–2D (see also Figure 1B).

Third, these measurements were linked to the parameters of the theory. Specifically, I assumed that the space constants of a given cloud 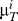 are proportional to the lengths of the semi-axes of the corresponding ellipse: 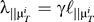 and 
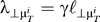
 , where *γ* is a certain dimensionless constant common to all axonal and dendritic clouds. 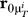 defined the displacement of the cloud center from the cell soma along the axis of symmetry. In addition, it was assumed that the synaptic densities at the cloud centers were the same for all four dendritic clouds and were described by a single parameter, 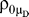 . Similarly, the synaptic densities at both axonal cloud centers were assumed equal and were described by a single parameter, 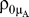 . Furthermore, since 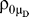 , 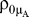 , and *δ* all enter only as their triple product into equation 12, defining the average number of contacts between a pair of axonal and dendritic clouds, they were merged into one parameter 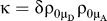 . The simplifying assumptions above imply that *κ* is a constant common to all types of axonal–dendritic cloud pairs. Thus, once the dimensions 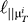 and 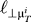 and positions 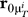 of individual clouds were estimated and fixed, the number of free parameters of the theory was effectively reduced to just two—*γ* and *κ*—that uniformly scaled, respectively, the spatial constants and the local magnitude of the synaptic density fields of all clouds.

Fourth, the parameters *γ* and *κ* were estimated by fitting theoretical curves for the average number of contacts 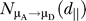 into corresponding empirical relationships 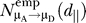 obtained by Hellwig [22]. 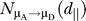 was calculated using the superposition principle for the synaptic density fields of the underlying clouds (assumption 2) and equations 11 and 12, defining the average number of contacts between individual cloud pairs. Two empirical curves, 
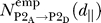
 and 
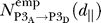
 , describing the intra-layer connections, were used to derive *γ* and *κ*. The resulting best fit (least squares) estimates were 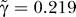 and 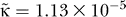 μm^−3^. Figure 2E and 2H shows the fitted (blue) and empirical (black) curves.

Finally, predictions of the theory were compared against independent experimental data. Specifically, the two remaining empirical curves, 
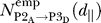
 and 
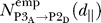
 , describing the inter-layer connections, were compared with the corresponding theoretical curves 
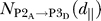
 and 
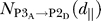
 that were calculated using 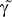 and 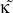 estimated from data on intra-layer connections. Figure 2F and 2G shows the predicted (red) and empirical (black) curves. To facilitate the comparison, theoretical data were also generated in the same representation as original experimental data [22] from which empirical relationships 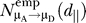 were obtained. Specifically, at each 1-μm increment of separation 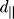 **,** the number of contacts was drawn from a Poisson distribution with the mean 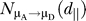 (see Theoretical Framework) independently 32 times, corresponding to 32 possible combinations of the reconstructed pre- and postsynaptic cell pairs of the same *μ*_A_ → *μ*_D_ type separated by the distance 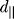 in [22], and then averaged and plotted in Figure 2E–2H as a dot.

One can see that overall there is very good agreement between the theory and experiment. Note that a single fixed pair of parameters 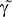 and 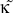 quantitatively explain a set of four different types of connections between two morphologically distinct types of pyramidal neurons. The theoretical average number of contacts matches fairly well to the experimentally determined ones in all four plots within the entire range of cell separation 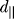 explored in [22]. In addition, the stochastic variations in the theoretical number of contacts (dots) are similar to variations seen in the corresponding experimental plots (cf. Figure 8A–8D of [22]).

#### Example 2: Clutch cells in L4 of cat visual cortex.

**Figure 3:**
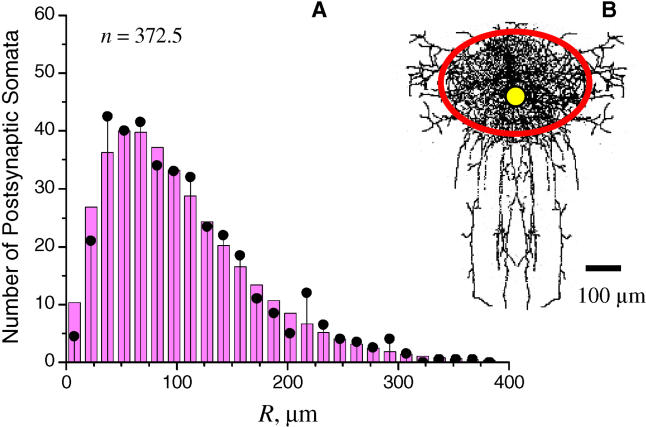
Somatic Connections of Clutch Cells in L4 of Cat Visual Cortex (A) Radial distribution of the average number of postsynaptic somata contacted by the axon. Dots with drop-lines show empirical distribution obtained by pooling data from the two cells [26]. Bars show theoretical distribution. (B) Image representing the average structure of the clutch cell axon. Yellow dot depicts cell soma. The ellipsoid captures the spatial extent of the synaptic cloud identified from the image. The dimensions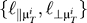 and the displacement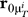 of the cloud were measured as illustrated in Figure 1B. The image was created using axonal arborization drawings based on data representations in [31] by kind permission of Z. Kisvárday.

In this example I use experimental data obtained by Kisvárday and colleagues [26,31], who studied local connections of clutch (a type of basket) cells in L4 of cat visual cortex. Most synaptic contacts formed by the axons of these types of inhibitory neurons are positioned at the postsynaptic cell soma and proximal dendrites [32]. Budd and Kisvárday [26] carried out a quantitative analysis in which they examined only the somatic connections. Based on an electron microscopy study [31], they assumed that all neuron somata (N_S_) opposed to boutons of clutch cell axons (C_A_) are contacted synaptically. Using previously 3D reconstructed axons of two clutch cells and recorded spatial locations of somata contacted by the axonal branches [31], they estimated the number of somatic connections C_A_ → N_S_ made by the individual clutch cell axon as a function of the radial distance *R* from the cell body. This was done simply by counting the number of contacted somata within a vertical cylindrical shell of a given radius *R* and a fixed width Δ*R,* centered at the clutch cell body, and traversing the entire depth of L4. The resulting two radial distributions, one for each cell, had very similar profiles (cf. Figure 3A and 3B of [26]), although the total numbers of counted postsynaptic somata were different (434 and 311).

To compare these experimental data with the theory, which provides ensemble averaged quantities, I first pooled data from the two cells and obtained the average observed radial distribution 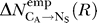 of somatic connections made by the clutch cell axons (Figure 3A, dots with drop-lines). The corresponding theoretical distribution 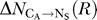 was calculated in the following way.

First, utilizing the assumption of cylindrical symmetry, an image representing the average spatial structure of the clutch cell axon was obtained. Specifically, individual drawings based on data representations in [31], depicting the projections of the 3D reconstructed axon on two (nearly frontal and sagittal) planes, were mirror reflected about the vertical axis. The underlying average image was then obtained by overlaying, as in example 1, the original and reflected drawings (Figure 3B).

Second, based on this image, the morphology of the clutch cell axon was described by a single cloud. The horizontal 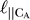 and vertical 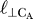 semi-axes of the corresponding enclosing ellipse were estimated from Figure 3B (see also Figure 1B). Since the analysis in Budd and Kisvárday [26] was restricted to L4 somatic connections only, the contribution from descending axonal branches projecting to deeper layers was disregarded.

Third, as in example 1, it was assumed that the axonal field space constants are proportional to the dimensions of the enclosing ellipse: 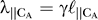 and 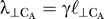 . Axonal–somatic connections were described using the same formalism as in the case of axonal–dendritic connections. Given that the volume of the postsynaptic soma is much smaller than the volume occupied by the clutch cell axonal ramifications, I assumed that the somatic field space constants 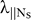 and 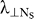 are small, such that 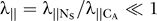 and 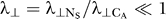 . In this case, the integrand in equation 12 can be expanded in the Taylor's series about 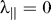 and 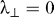 ; retaining only the free term, I obtained the average number of contacts 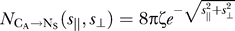 made by the clutch cell axon with the cell soma located at 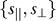 , where 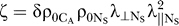 is a dimensionless parameter, and 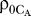 and 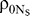 are the synaptic densities at the corresponding cloud centers. Integrating 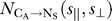 over the volume of a cylindrical shell of radius *R* and width Δ*R,* I obtained the underlying radial distribution of somatic connections:


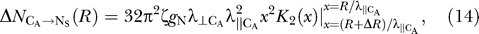


where *K*_2_(*x*) is the modified Bessel function of order 2, and *g*_N_ is the neuronal density in L4, which was set to 5.4 × 10^4^ mm^−3^ (cf. [26]). The theoretical distribution is parameterized by *ζ* and *γ*. The former scales the overall amplitude of the distribution whereas the latter uniformly scales the spatial constants 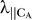 and 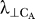 , which, in turn, shape the radial profile of the distribution.

Finally, the parameters *γ* and *ζ* were varied in order to bring the theoretical distribution 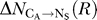 into a correspondence with the experimentally obtained distribution 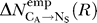 . The values of the adjusted parameters were 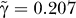 and 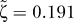 ; the resulting theoretical distribution is shown in Figure 3A (bars). One can see that the theory captures the features of the experimental distribution adequately. Particularly, the profile of 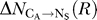 matches very well with the profile of 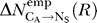 in the whole range of *R*. Note also that the value of 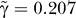 derived for the somatic connections of the clutch cell axons is very close to the value of 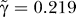 derived for the connections between the pyramidal neurons in L2/L3.

#### Example 3: Pyramidal neurons in L5 of rat somatosensory cortex.

**Figure 4:**
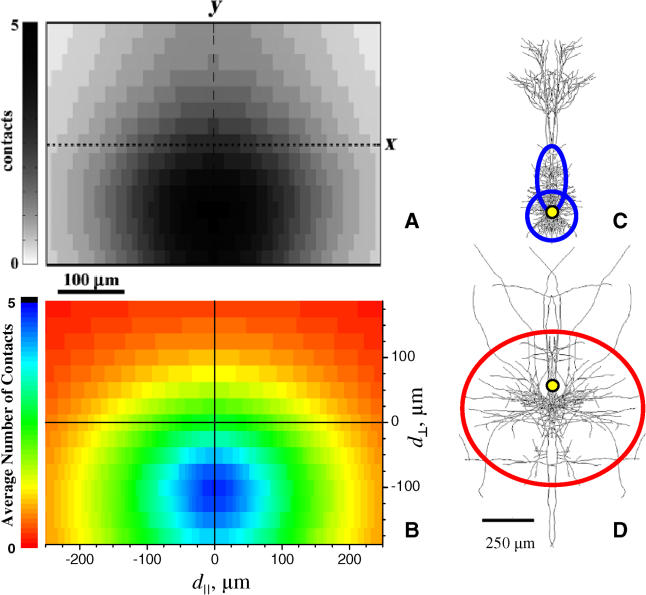
Connectivity between Pyramidal Neurons in L5 of Rat Somatosensory Cortex (A and B) Connectivity map showing the average number of contacts formed between the presynaptic cell positioned at the origin and the postsynaptic cell at location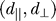 : (A) empirical map adapted from data representations in [23] by kind permission of H. Markram and G. Silberberg; (B) theoretical map. (C and D) Images representing the average structures of dendrites (C) and axons (D) of pyramidal neurons in L5. Yellow dots depict cell somata. Ellipsoids capture the spatial extent of the synaptic clouds identified from these images. The dimensions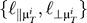 and the displacement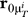 of each cloud were measured as illustrated in Figure 1B. The images were created using dendritic and axonal arborization drawings based on data representations in [23] by kind permission of H. Markram and G. Silberberg.

This example, illustrated in Figure 4, relies on work of Markram and colleagues [23] in which physical connectivity between pyramidal neurons in L5 of rat somatosensory cortex was estimated based on the 3D reconstructed morphology of 11 axons (P5_A_) and 14 dendrites (P5_D_). The key idea of their approach is that statistics of cell arbors could be used to estimate the average number of P5_A_ → P5_D_ axonal–dendritic contacts formed by a pair of neurons as a function of their relative locations. Unlike earlier work of Hellwig [22], considered in example 1, who explicitly averaged the number of physical contacts over pairs of reconstructed axons and dendrites positioned at a given relative distance, Kalisman et al. [23] first averaged the geometry of ramifications over reconstructions of single arbors, separately for the axons and dendrites, to obtain two maps, called the axonal and dendritic templates. Then, using these empirical templates, the connectivity map 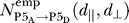 **—**the average number of contacts formed by a pair of neurons as a function of their horizontal, 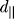 , and vertical, 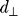 , separation—was estimated (Figure 4A).

To compare these results with my theoretical model, I first visualized, as in previous examples, the average spatial structures of the underlying arbors using drawings based on data representations in [23]. The resulting two images are shown in Figure 4C and 4D. The morphology of P5_A_ axons was described by a single cloud corresponding to extensively branching collaterals below the cell soma and was represented by an oblate ellipsoid (Figure 4D). The structure of P5_D_ dendrites was described by two clouds corresponding to basal and oblique apical ramifications represented by a sphere and a prolate ellipsoid, respectively (Figure 4C). The apical tuft dendrites that ramify in the upper cortical L1 and L2 were disregarded because, within the range of the vertical separation 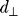 explored in [23], the tuft dendrites nearly do not overlap with the axon collaterals and, therefore, their contribution to the connectivity can be neglected. Thus, altogether, three clouds were used to describe L5 pyramidal neuron morphology. For each cloud 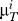 (*i* = 1 for *μ*_A_ = P5_A_ and *i* = 1,2 for *μ*_D_ = P5_D_) the horizontal, 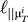 , and vertical, 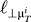 , semi-axes of the corresponding ellipse as well as the position of its center relative to the cell soma, 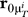 , were estimated from the drawings in Figure 4C and 4D (see also Figure 1B).

The theoretical average number of contacts 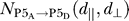 was calculated in the same way as explained in example 1. As before, I assumed that the space constants of individual clouds are given by 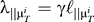 and 
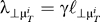
 . Since the optimal values of the parameter *γ* derived in two previous examples were very close (0.207 and 0.219), in this case I simply set *γ* = 0.215. Thus *κ* (see example 1) was the only remaining free parameter. The value of *κ* = 2.10 × 10^−5^ μm^−3^ was determined by normalizing the amplitude of the peak in the connectivity map 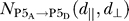 to 4.8 contacts, in accordance with Kalisman et al. [23]. One can see that the resulting map, shown in Figure 4B, is in good quantitative agreement with the empirical map 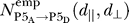 **,** shown in Figure 4A. Particularly, the peak of theoretical connectivity occurred at 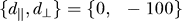 μm, when the postsynaptic neuron was located 100 μm below the presynaptic cell soma, as was also observed in the empirical map 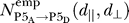 . The connectivity at the map origin, which could be considered as the average number of contacts between the axon and dendrite of the same neuron, i.e., the average number of autaptic contacts, was 2.7. This is in good agreement with the 2.3 ± 0.9 autapses per neuron estimated by Lübke et al. [28] from detailed light and electron microscopy study of the same type of neurons, and very close to the 2.8 contacts reported by Kalisman et al. [23] for the empirical map 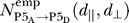 .

## Discussion

In this work I proposed a simple theoretical model of local synaptic connectivity between a pair of cortical neurons that takes into account the morphological structure of axons and dendrites and the relative spatial locations of the pre- and postsynaptic somata. To understand the implications of the underlying simplifying assumptions, the theoretical number of synaptic contacts was compared with the number of contacts estimated empirically in quantitative studies of synaptic connectivity [22,23,26]. In these studies 2D drawings of arbors (necessary for extracting the phenomenological parameters of the theory) and empirically estimated numbers of contacts at various separations between pre- and postsynaptic cells (required for the comparison against the predictions of the theory) were both published. In all three examples considered there was very good agreement between the theory and experiment, within a wide range of pre- and postsynaptic cell separations.

The present approach relies on the assumption that the interactions between axons and dendrites are negligibly small and, therefore, their morphological properties can be treated independently. This is adequate, particularly, for the axons of pyramidal cells that form nonspecific axonal–dendritic contacts (examples 1 and 3). In addition, I demonstrated that the same formalism can be extended to the case of highly specific contacts such as somatic synapses formed by the axons of inhibitory interneurons (example 2), and thus the present approach, unlike the previously suggested method [23], is able to quantitatively predict this type of synaptic connectivity as well. These results suggest that the theoretical framework and the chosen functional form for the synaptic density fields of axons and dendrites effectively capture 3D morphologies of a variety of neurons (GABAergic and pyramidal, from different cortical layers, areas, and organisms) and describe the two types of synaptic connectivity (excitatory axonal–dendritic and inhibitory axonal–somatic) between cell pairs fairly well. It remains to be seen, however, whether this approach will be able to produce satisfactory results for other types of neurons.

An important aspect of the theoretical framework is the “linearization” of the complex structure of an axonal/dendritic arbor of a given morphological type *μ_T_,* representing it as a linear combination of basic structures—elementary synaptic clouds (see assumption 2). It was demonstrated that by measuring the horizontal 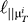 and vertical 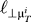 spread of individual cloud ramifications observed in the 2D drawings of the underlying arbors, one can estimate the corresponding space constants 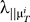 and 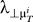 , assuming a linear isotropic relationship between the physical 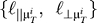 and characteristic 
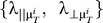
 sizes of the cloud: 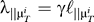 , 
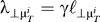
 . The central result of this paper is that the scaling parameter *γ* providing the best correspondence between theory and experiment had a nearly constant value regardless of the morphological origin of synaptic clouds considered in the examples. Indeed, *γ* varied within a narrow range (0.207 < *γ* < 0.219) and, therefore, appears to be nearly independent of the type of originating arbor (axonal or dendritic), neuron (pyramidal or GABAergic), cortical layer (superficial L2/L3 or deep L5), cortical area (somatosensory or visual), and organism (rat or cat). As a result, it is tempting to think of *γ* as a kind of “universal” space calibration constant that translates the physical dimensions of any given synaptic cloud into the space constants describing properly the spatial distribution of the underlying cloud synaptic density. Further quantitative studies, however, are necessary to find out whether *γ* is truly invariant with respect to the whole multitude of diverse neuron morphologies observed in the cortex.

In the present consideration it was assumed that the parameter 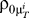 , which defines the density at the center of a particular cloud 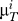 , is constant for all constituent clouds of the underlying arbor *μ_T_*: 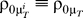 . In this case, the theoretically predicted average number of contacts between a pair of neurons is affected, in fact, by the product of two such parameters, one for the presynaptic and the other for the postsynaptic arbor. Therefore, regardless the number of pre- and postsynaptic clouds involved, as far as the synaptic connectivity is concerned, a single parameter incorporating this product (*κ* in examples 1 and 3; *ζ* in example 2) is what matters. It defines the amplitude scale of synaptic interactions (i.e., the peak number of contacts) between pairs of neurons, in contrast to *γ,* which defines the spatial scale of such interactions (i.e., how fast the number of contacts decreases as the separation between neurons increases). Unlike *γ,* which turned out to be nearly constant, the value of *κ* varied substantially in the examples considered. If one is interested in the absolute number of synaptic contacts, then this parameter should be calibrated for each morphologically distinct type of neuronal pair by comparing the predicted number of synaptic contacts with the experimentally measured one at known cell separations. The main value of the present theoretical framework, however, is in the determination of the relative scaling of the number of synaptic contacts between a pair of cells with their spatial separation, rather than the exact number of contacts.

It is noteworthy that although the number of 3D reconstructed neurons is growing, the existing empirical methods [22,23] for the estimation of synaptic connectivity, which are based on 3D reconstructions, are cumbersome. In contrast, the present theoretical framework, although less accurate than the methods presented in [22,23], provides a straightforward approach for estimating synaptic connectivity by (i) extracting the relevant structural parameters of axons and dendrites from 2D arborization drawings, and then (ii) plugging them into a compact analytical expression providing the number of synaptic contacts as a function of relative cell positions.

The significance of this work, however, goes beyond the derivation of an analytical expression describing synaptic connectivity between morphologically distinct neuronal pairs. For example, the present approach could be used for deciphering the structure of local synaptic circuitry (i.e., the pattern of connections between neurons) in a cortical region of interest. In particular, one could estimate the individual contributions from diverse types of neurons distributed across cortical layers to the net synaptic input received by a neuron of a given type *μ*_D_. The number of synaptic contacts contributed by all presynaptic neurons of a particular type *μ*_A_ could be obtained by integrating the theoretical number of contacts formed between the pair *μ*_A_→*μ*_D_ over the positions of the presynaptic neurons *μ*_A_ in the underlying cortical region. Note that such an approach accounts for both specific morphologies of neurons and spatial distributions of neurons. These are important factors for the quantification of local synaptic circuits because the densities of morphologically distinct types of neurons vary across cortical layers in a specific fashion [2]. Also, vertically oriented anatomical minicolumns, clearly visible in certain cortical areas [2], introduce an additional order in the cortical distribution of neurons. In general, such a local spatial ordering of neurons could further structuralize cortical circuitry and contribute to the formation of functional modules (such as cortical columns) with sharp borders [33]. The theoretical predictions of local connectivity patterns can be compared with independent quantitative experimental studies of cortical synaptic circuits.

Recently, Stepanyants, Hof, and Chklovskii [34] provided an elegant and insightful analysis of information storage capacity associated with local structural plasticity, without major remodeling of dendritic or axonal arbors. The key aspect of their approach is the calculation of the number of potential synapses that a given dendrite can form with axons passing within the spine length from the dendrite. The capacity for altering connectivity patterns through formation and elimination of synapses made by dendritic spines—the information storage capacity—was then characterized in terms of the filling fraction *f—*the ratio of actual to potential synapses.

The framework of potential synapses could be also used in different contexts, providing insights into different aspects of synaptic connectivity. In the present approach the specificity of synaptic connections is determined by geometrical factors such as the layout of axonal and dendritic branches and the relative spatial positions of pre- and postsynaptic cells. Can the specificity of synaptic connections go beyond the geometry, without major remodeling of dendritic or axonal arbors? This is possible if the number of potential synapses as defined in [34] is greater than the number of actual synapses. The specificity could be achieved by selecting the appropriate subset from the pool of potential synapses. In this interpretation, the filling fraction characterizes the capacity to form specific synaptic connections apart from the geometrical factors considered above. Using estimates of spine length from several brain areas, Stepanyants et al. [34] calculated the filling fraction *f* and found that the information capacity ranges from three to four bits per synapse of pyramidal neuron. In the context of synaptic specificity this implies that, on average, each presynaptic site can choose its postsynaptic partner roughly from three to four available sites, without major remodeling of axonal or dendritic arbors.

Is this potential for pyramidal neuron local synaptic specificity actually realized in the cortex? Until a short time ago, this was an open question. In a recent paper, Kalisman, Silberberg, and Markram [35], using confocal microscopy and whole-cell recordings from pairs and triplets of thick tufted L5 pyramidal neurons of rat somatosensory cortex, found that axons physically contact neighboring dendrites without any bias. This is consistent with the present theoretical model as well as previous studies [22,23]. The average number of axonal–dendritic touches between synaptically connected pairs of neurons was 6.6 ± 1.5. However, only 1.5 ± 0.3 of those touches were characterized as bouton–spine contacts (putative synapses). Special analysis carried out in [35] strongly suggested that the bouton–spine contacts were indeed synapses. Thus, Markram and colleagues [35] demonstrated that indeed only a small fraction of potential synaptic sites (touches) are realized as actual synapses (bouton–spine contacts). One can think that the conversion of a potential to actual synapse is a random, stochastic process, i.e., a given touch is transformed into a synapse with a certain probability. However, do these conversions of potential synapses occur independently from each other (i.e., uniformly randomly) or in a specifically coordinated fashion (i.e., nonuniformly randomly)? In a recent study, Chklovskii and colleagues [36] probed synaptic connections using quadruple whole-cell recordings from L5 pyramidal neurons in rat visual cortex. Statistical analysis of several hundred such simultaneous recordings revealed that reciprocal synaptic connections as well as several three-neuron connectivity patterns are more common than one would expect in uniformly randomly wired quadruplets. This study, therefore, suggests that presynaptic sites can select their partners from the pool of potential postsynaptic sites in a specific way. Fine-scale specificity has been also reported by Callaway and colleagues [37] in rat visual cortex for connections between adjacent pyramidal neurons in L2/L3 forming a group of selectively interconnected neurons that receive common excitatory input from L4.

Thus, the specificity in synaptic connectivity without major remodeling could occur at least at two levels. While the geometry of axons and dendrites and relative cell positions define the coarse level of specificity, recent work [35–37] suggest that the fine-tuning of synaptic connectivity in local microcircuits could be achieved by selecting an appropriate subset from the pool of potential synapses. The present theoretical framework considers the coarse specificity only. As soon as sufficient data become available, new quantitative models accounting for the fine-tuning of specificity in local cortical circuits should be developed.

In conclusion, the phenomenological approach to local synaptic connectivity described in this paper provides a remarkably simple way for extracting the relevant structural parameters of axons and dendrites from 2D arborization drawings. It was demonstrated that a crude approximation of axonal and dendritic arbors as a superposition of a set of ellipsoids is satisfactory for the purpose of quantitative estimation of synaptic connectivity between specific types of neurons as a function of their relative locations. Since for many types of neurons 2D drawings are available from literature, the present approach could be of principal significance for the practicality of deciphering synaptic microcircuits of a given cortical region based on the actual observed densities of specific types of neurons and their morphologies. It could also have significant implications for computational models of cortical networks by making it possible to wire up simulated neural networks in a realistic fashion.

## Materials and Methods

### 

#### Evaluation of the number of contacts between a pair of elementary clouds.

The aim of this section is to explain how I evaluated the average number of synaptic contacts 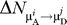 formed between the axonal cloud 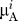 and the dendritic cloud 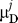 separated by the displacement vector Δ**r** connecting their centers. Let us place the origin of a Cartesian coordinate system at the center of the cloud 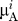 , so that the *z*-axis is parallel to the clouds' axes of cylindrical symmetry. Using synaptic density fields given by equation 10, and introducing the dimensionless variables (equation 11), equation 9 defining the average number of synaptic contacts can be rewritten as


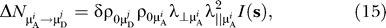


where


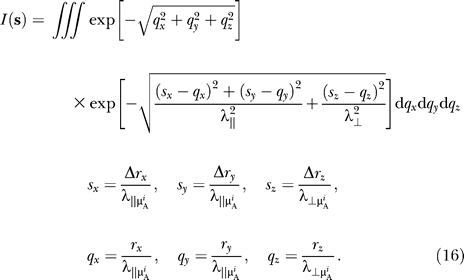


Note now that the above integral is a convolution of two functions: *I*(**s**) = *g* * h, where


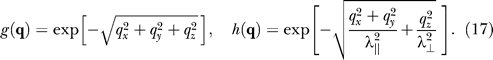


To evaluate *I*(**s**), one can first find its Fourier transform 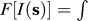 *I*(**s**)*e* ^−*i*ks^d^3^ **s** using the property that the transform of a convolution is the product of the Fourier transforms of the factors of the convolution: 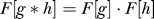 . The transform of *g*(**q**) is given by (e.g., [38])


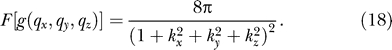


Next, note that 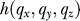 = 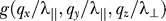 . Applying the scaling property of the Fourier transform, one obtains


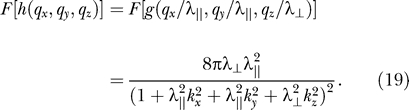


*I*(**s**) can be then found by the inverse transformation:


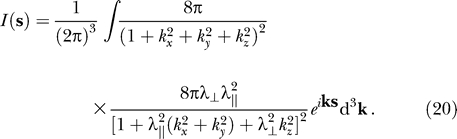


Exploiting the symmetry present in the problem, let us change to conventional cylindrical coordinates:


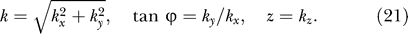


The integral (equation 20) can be then rewritten as


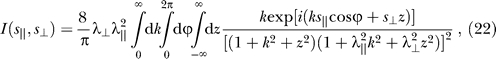


where 
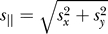
 and 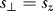 (cf. equation 11). Integrating over *ϕ*, one obtains


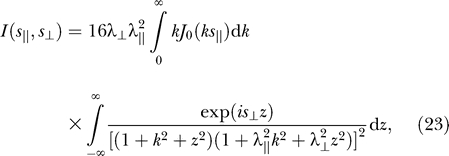


where *J*_0_(*x*) is the Bessel function of order zero (e.g., [38]). To integrate over *z,* one can use the method of contour integration (e.g., [39]) provided by the theorem of residues from the theory of functions of a complex variable. The integrand has two poles of order two in the upper-half of complex *z*-plane. Computing the residues at these poles and choosing the large semicircle in the upper-half plane as the contour of integration, one obtains the final result (equation 12).

##### Special case of similar shapes.

Consider a case 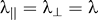 , in which interacting axonal and dendritic clouds have similar, but not necessarily spherical, shapes (cf. equation 11). Equation 20 then becomes


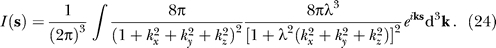


Using now the higher symmetry of this case, one can change to spherical coordinates:


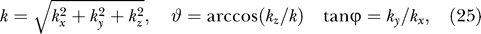


and, after integrating over *ϕ*, obtain


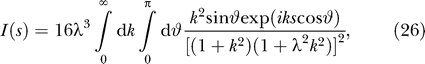


where


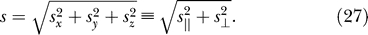


Making the change of variable 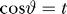 *,* the double integral (equation 26) can be further simplified and reduced to a one-dimensional integral:


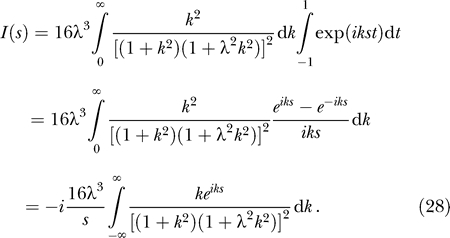


This last integral is evaluated using the same method of contour integration as in the general case considered above. Again, the integrand has two poles of order two in the upper-half of complex *k*-plane. Computing the residues at these poles and choosing the large semicircle in the upper-half plane as the contour of integration, one obtains equation 13.

## Abbreviations

2D: two-dimensional
3D: three-dimensional
L[number]: layer [number]
